# The characterization of extracellular vesicles-derived microRNAs in Thai malaria patients

**DOI:** 10.1186/s12936-020-03360-z

**Published:** 2020-08-10

**Authors:** Nutpakal Ketprasit, Iris Simone Cheng, Fiona Deutsch, Nham Tran, Mallika Imwong, Valery Combes, Duangdao Palasuwan

**Affiliations:** 1 Graduate Programme in Clinical Hematology Sciences, Department of Clinical Microscopy, Faculty of Allied Health Sciences, Chulalongkorn University, Bangkok, Thailand; 2 Malaria and Microvesicles Research Group, School of Life Sciences, Faculty of Sciences, University Technology of Sydney, Ultimo, Sydney, NSW 2007 Australia; 3 Non-coding RNA Cancer Group, School of Biomedical Engineering, Faculty of Engineering and IT, University Technology of Sydney, Sydney, NSW Australia; 4 Department of Molecular Tropical Medicine and Genetics, Faculty of Tropical Medicine, Mahidol University, Bangkok, Thailand; 5 Oxidation in Red Cell Disorders Research Unit, Department of Clinical Microscopy, Faculty of Allied Health Sciences, Chulalongkorn University, 154 Rama 1 Road, Pathumwan, Bangkok, 10330 Thailand

**Keywords:** Malaria, Patients, *Plasmodium falciparum*, *Plasmodium vivax*, Extracellular vesicles, microRNAs

## Abstract

**Background:**

Extracellular vesicles (EVs) have been broadly studied in malaria for nearly a decade. These vesicles carry various functional biomolecules including RNA families such as microRNAs (miRNA). These EVs-derived microRNAs have numerous roles in host-parasite interactions and are considered promising biomarkers for disease severity. However, this field lacks clinical studies of malaria-infected samples. In this study, EV specific miRNAs were isolated from the plasma of patients from Thailand infected with *Plasmodium vivax* and *Plasmodium falciparum*. In addition, it is postulated that these miRNAs were differentially expressed in these groups of patients and had a role in disease onset through the regulation of specific target genes.

**Methods:**

EVs were purified from the plasma of Thai *P. vivax*-infected patients (n = 19), *P. falciparum*-infected patients (n = 18) and uninfected individuals (n = 20). EV-derived miRNAs were then prepared and abundance of hsa-miR-15b-5p, hsa-miR-16-5p, hsa-let-7a-5p and hsa-miR-150-5p was assessed in these samples. Quantitative polymerase chain reaction was performed, and relative expression of each miRNA was calculated using hsa-miR-451a as endogenous control. Then, the targets of up-regulated miRNAs and relevant pathways were predicted by using bioinformatics. Receiver Operating Characteristic with Area under the Curve (AUC) was then calculated to assess their diagnostic potential.

**Results:**

The relative expression of hsa-miR-150-5p and hsa-miR-15b-5p was higher in *P. vivax*-infected patients compared to uninfected individuals, but hsa-let-7a-5p was up-regulated in both *P. vivax*-infected patients and *P. falciparum*-infected patients. Bioinformatic analysis revealed that these miRNAs might regulate genes involved in the malaria pathway including the adherens junction and the transforming growth factor-β pathways. All up-regulated miRNAs could potentially be used as disease biomarkers as determined by AUC; however, the sensitivity and specificity require further investigation.

**Conclusion:**

An upregulation of hsa-miR-150-5p and hsa-miR-15b-5p was observed in *P. vivax*-infected patients while hsa-let-7a-5p was up-regulated in both *P. vivax*-infected and *P. falciparum*-infected patients. These findings will require further validation in larger cohort groups of malaria patients to fully understand the contribution of these EVs miRNAs to malaria detection and biology.

## Background

According to the World Health Organization (WHO) World Malaria Report 2019, there were an estimated 405,000 deaths, and 228 million cases were reported globally [1]. *Plasmodium vivax* has the highest prevalence and is known to cause relapse infection [2], whereas *P. falciparum* is the most virulent species causing severe syndromes. Malaria appears to be more devastating worldwide as the emergence of artemisinin-resistant malaria parasites [3, 4]. To overcome the disease, novel drug development is essential. On the other hand, in-depth understanding of the parasite biology and mechanisms underlying the disease is also urgently needed.

Extracellular vesicles (EVs), which are small membrane-bound vesicles, have been explored in malaria for the last decade. Classification of EVs is based on their cellular origin, size, and biological functions [5]. Two major types of EVs have been studied broadly including microvesicles (previously named microparticles) and exosomes. Another type of EVs are the apoptotic bodies which are released during the apoptotic process [6]. Microvesicles are vesicles that bleb from the cell membrane whereas exosomes are released from multivesicular bodies (MVBs) through an exocytotic process in the endolysosomal pathway [7].

Typically, EVs can be detected in very low numbers in healthy individuals. However, upon activation which is triggered by various pathological conditions [8–11], the number of EVs present in biological fluids increases. Various biomolecules were identified in EVs such as proteins, lipids, and nucleic acids [12]. Such biomolecules entrapped in EVs play many important roles in intercellular communication in numerous diseases, including malaria [13, 14].

EVs numbers have been shown to increase during malaria infection both in patients and in experimental malaria models [10, 11, 15–18]. They are associated with either protective [19] or enhanced pathogenicity of malaria infection [11, 16–18, 20, 21]. The most common feature demonstrated in malaria is that EVs can act as immunomodulators. EVs from infected-erythrocytes can stimulate innate immune cells including macrophages [22], natural killer cells [23], monocytes and neutrophils [24]. Interestingly, EVs participate in cell–cell communication between parasites and parasites or host cells. Exosome-like vesicles have played a role in gametocytogenesis, which is crucial for malaria transmission, and these small membrane-bound vesicles could transfer drug-resistance markers to drug-sensitive parasites [25]. Additionally, EVs which cargo *P. falciparum* lactate dehydrogenase (PfLDH) could control parasite density in vitro [26]. One study also showed that EVs enriched with the parasite’s genomic DNA, released from infected red blood cells could be internalized by monocytes and elicited an innate immune response [27].

As mentioned above, EVs carry many kinds of biomolecules including microRNAs (miRNAs). EVs were shown to protect the miRNAs from RNases-mediated degradation and EVs-entrapped miRNAs were also demonstrated to have regulatory functions [28–30]. MiRNAs are members of the non-coding RNAs family which were first discovered in *Caenorhabditis elegans* [31]. Malaria parasites do not express specific miRNAs [32, 33], as Dicer or Argonaut encoding genes are not found in these parasites [34–36]. However, several studies showed that miRNAs could translocate to *P. falciparum* [37, 38]. A pioneer investigation by Lamonte et al. showed interaction between human miRNAs and *P. falciparum*, where the expression of hsa-miR-451a and hsa-let-7i were increased in sickle cells. These miRNAs could inhibit parasitic protein translation resulting in low proliferation of parasites [38]. In other studies, expression of miRNAs in heart and brain tissues from mice with cerebral malaria were shown to be dysregulated when compared to non-cerebral malaria [39, 40].

MiRNAs, human Argonaute protein and RISC complex could be detected in the parasites [37] as well as in EVs released by infected erythrocytes [41, 42]. EVs carried Argonaut-miRNAs complex affecting recipient cells that have been demonstrated to alter vascular function [41] or the parasite’s *var* gene expression [43]. These studies strengthen the roles of EVs-derived miRNAs in malaria pathogenesis. There are only three studies that have analysed miRNAs in malaria-infected patients. In human plasma samples, the expression of hsa-miR-451a and hsa-miR-16 in *P. vivax*-infected patients were lower than those in *P. falciparum*-infected patients. Moreover, hsa-miR-451a and hsa-miR-16 had a negative relationship with parasitaemia [44]. In a post-mortem study, the expression of various miRNAs were found to be differentially expressed between malaria and non-malaria deaths [45]. Furthermore, hsa-miR-146a was newly predicted to play a role in innate immunity in pregnancy malaria [46]. Taken together, these studies highlight the potential roles of miRNAs in malaria infection.

Despite the fact that the presence of EVs-derived miRNAs has been demonstrated both in vitro and in experimental cerebral malaria [47], according to the literatures available, no study has analysed EVs-derived miRNAs in human plasma. Thus, this present study examined the relative expression of selected miRNAs isolated from human plasma EVs. In the present study, the relative expression among three biological groups was compared, i.e., *P. vivax*-infected or *P. falciparum*-infected patients and uninfected individuals. The whole population of circulating EVs, namely microvesicles together with exosomes were analysed to gain a better understanding of the whole EV-bound miRNA population and to not restrict this analysis to one sub-population. By studying the EV compartment as a whole the chances of finding differences in miRNA expression between the groups would be increased. Five miRNA were selected based on previous in vitro, animal model, or clinical studies that suggested these miRNAs had a potential involvement in malaria, namely: hsa-miR-451a, hsa-miR-150-5p, hsa-miR-15b-5p, hsa-let-7a, and hsa-miR-16-5p [37–39, 41–44, 48]. This study provides novel insight about circulating EVs-derived miRNAs in human malaria.

## Methods

### Human samples collection and preparation

Malaria patients with *P. falciparum* or *P. vivax* were confirmed by microscopic examination by medical laboratory scientists at Buntharik district hospital in Ubon Rachathani. (Ethics approval by Ethics committee of the Faculty of Tropical Medicine, Mahidol University, MUTM 2012-046-05). Parasite species were identified and parasitaemia of each sample was calculated by counting the number of malaria-infected erythrocytes over the number of normal erythrocytes per thousand cells. Uninfected donors were recruited, and venipuncture was performed for all donors using tri-potassium ethylenediaminetetraacetic acid (K3EDTA) as an anticoagulant. K3EDTA blood samples were then centrifuged at 1500*×g* for 15 min.

Then, platelet-free plasma samples were prepared by centrifugation at 13,000*×g* for 2 min at room temperature (RT) [17]. Supernatants were collected and stored at − 80 °C for further isolation of EVs. Samples were then shipped to the University of Technology Sydney where all experiments were performed.

### Extracellular vesicles isolation from human plasma

500 µL of supernatants were mixed with 200 µL of sodium citrate and 300 µL of phosphate buffered saline (PBS) and then centrifuged at 150,000*× g* for 3 h at 15 °C. Supernatants were discarded and pellets were resuspended in 100 µL sodium citrate and 900 µL PBS and subjected to centrifugation at 150,000*×g* for 3 h at 15 °C.

### Total RNA extraction

Pellets obtained after centrifugation were homogenized with 1 mL of RNAzol (Molecular Research Center, Inc) and 400 µL of UltraPure™ DNase/RNase-Free distilled water (Invitrogen ™). After a five-minute incubation at RT, they were centrifuged for 15 min at 12,000*×g* at 4 °C. Supernatants were transferred to new tubes with 800 µL isopropanol and 5 µL glycogen (5 mg/mL) then gently mixed. They were then placed at − 30 °C overnight and spun for 10 min at 12,000*×g* at 4 °C the next day. Pellets were kept and washed with cold 75% ethanol to remove excess isopropanol, spun at 9000*×g* for 3 min at 4 °C, the supernatant was discarded, and these steps were repeated twice. Later, excess ethanol was removed by spinning at 12,000*×g* for 30 s. RNA pellets were resolubilized with 10 µL of UltraPure™ DNase/RNase-Free distilled water and heated for 5 min at 55 °C. Lastly, they were vortexed and briefly spun down.

### Total RNA concentration measurement and cleaning

RNA concentration was measured using the NanoDrop One Microvolume UV–Vis Spectrophotometer (Thermo Scientific™). If the quality of total RNA in the samples was not optimal, i.e., the Nanodrop flagged the result as phenol contamination or Absorbance 260/230 < 1.7, a sodium acetate (NaOAc), a cleaning step was performed. Briefly, 5 µL of glycogen (5 mg/mL) and 2.5 µL of sodium acetate (3 M pH5.5) were added to samples and mixed comprehensively. The mixture was supplemented with 110 µL of absolute ethanol and incubated overnight in − 30 °C. Later, washing steps were performed as mentioned in the RNA extraction with RNAzol above.

### Primers for miRNAs detection

All primers in this study was purchased from Thermo Fisher Scientific.

Five human miRNAs were used to perform quantitative polymerase chain reaction as described in Table 1.

**Table 1:** Primers used for quantitative PCR

miRNAs	NCBI accession number	Mature miRNAs sequences
hsa-miR-451a	MI0001729	AAACCGUUACCAUUACUGAGUU
hsa-miR-15b-5p	MI0000438	UAGCAGCACAUCAUGGUUUACA
hsa-miR-16-5p	MI0000070	UAGCAGCACGUAAAUAUUGGCG
hsa-let-7a-5p	MI0000060	UGAGGUAGUAGGUUGUAUAGUU
hsa-miR-150-5p	MI0000479	UCUCCCAACCCUUGUACCAGUG

### Reverse transcriptase quantitative polymerase chain reaction (RT-qPCR)

As the NanoDrop was not sensitive enough to measure the RNA concentration, fixed volumes of RNA for cDNA synthesis were used. To analyse miRNAs expression, 0.5 µL of total RNA was used for cDNA synthesis. RT-qPCR was performed as per manufacturer protocol using TaqMan^®^ fast advanced master mix and TaqMan^®^ advanced miRNA assay. Quantitative PCR (qPCR) was run on the QuantStudio 6 flex system (Applied Biosystem) in triplicate together with distilled water as negative control.

### Relative expression analysis

Only samples that had not been already freeze-thawed were included in the study to avoid RNA degradation. Comparative threshold cycle or quantification cycle C_q_ (as described in [49]) method was used to analyse the qPCR data. Each sample was run in triplicates. Then, the average C_q_ from the three C_q_ values of those samples was calculated. To select one miRNA as an endogenous control, mean, standard deviation and variance for each miRNA analysed were calculated. ΔC_q_ was then calculated using the following equation ΔC_q_ = C_q_ of miRNA − C_q_ of endogenous control and relative expression was calculated by 2^(−ΔΔCq)^ [50].

### Target prediction and pathway involvement of dysregulated miRNAs

To predict possible targets of up-regulated miRNAs, the miRNAs of interest was submitted to Targetscan Release 7.2 and the predicted target genes were retrieved [51]. Then, the targets that overlapped genes involved in malaria pathway were obtained from the Kyoto Encyclopedia of Genes and Genomes (KEGG) [52]. In order to identify the pathways for individual and combined analysis of miRNAs, DIANA-mirPath v3.0 was used, with a 5% false discovery rate (FDR) [53]. Fisher’s exact test (Hypergeometric distribution) was applied for enrichment analysis. The potential pathways were considered based on their *p* value (p < 0.05).

### Evaluation of potential EVs-derived miRNA as biomarker

Mean of Delta Cq for each miRNA was used in this analysis following the previous study [54]. The receiver operating characteristic (ROC) was calculated to propose the potential use of miRNAs as diagnostic tools. Area under the curve (AUC) was analysed to show the accuracy of the test with p-value of each analysis.

### Statistical analysis

For the samples, demographic, mean age of each group was presented by average and standard deviation. All data was tested for their normality by using Kolmogorov–Smirnov or Shapiro–Wilk tests and a non-parametric statistical analysis method was chosen. Comparison of parasitaemia percentage was achieved by the Mann–Whitney U Test. Kruskal–Wallis test was used for comparison of relative expression of miRNAs in each biological group followed by post hoc analysis using Dunn’s test. Statistical significance for all tests was considered significant for α = 0.05. All statistical tests were analysed by Prism 8. All figures shown in this article were created by Prism 8 software for Mac, GraphPad Software, La Jolla California USA, https://www.graphpad.com.

## Results

### Patient’s characteristics

**Fig. 1:**
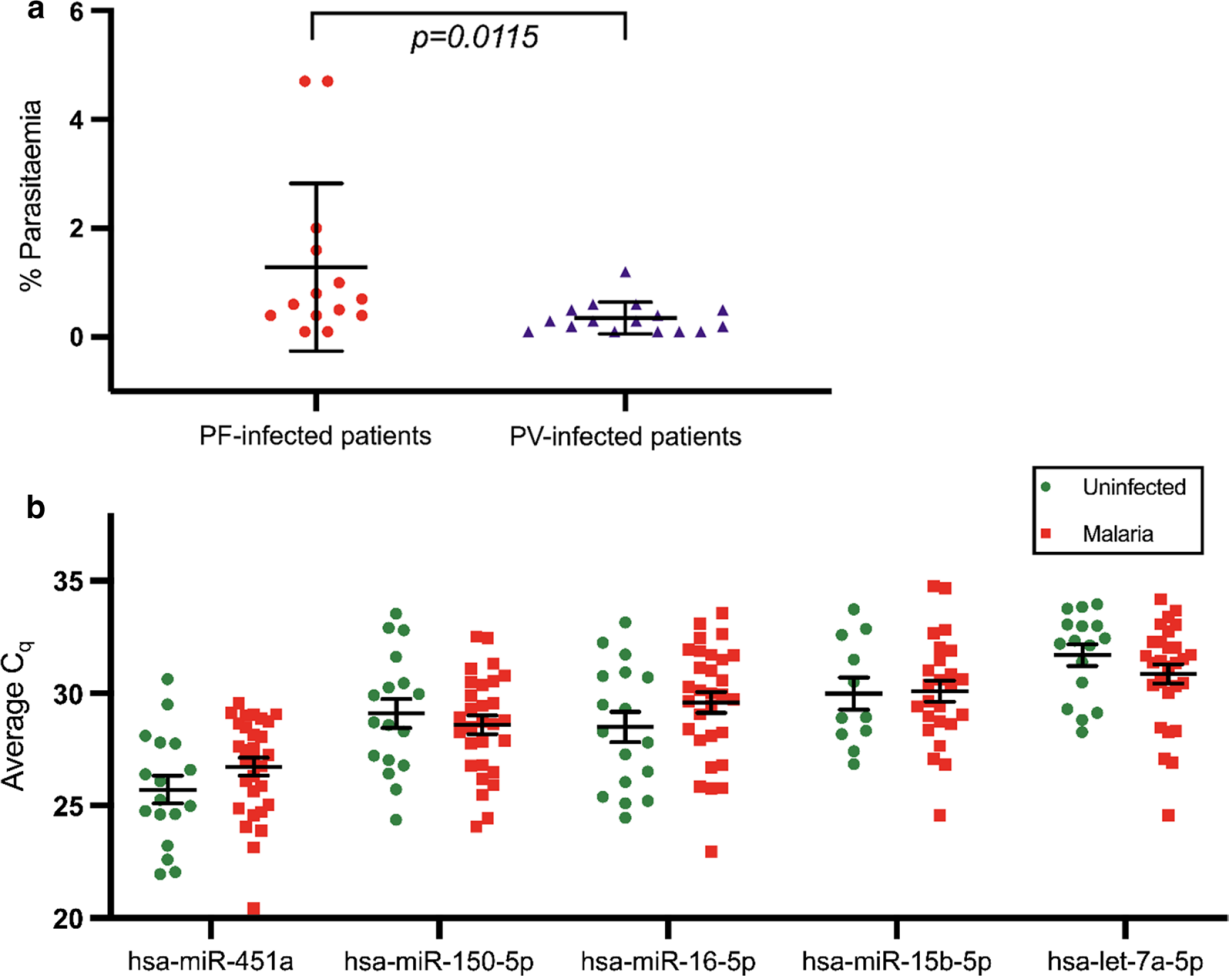
**a** Malaria infected patients with *P. vivax* or *P. falciparum* calculated as a percentage of parasitaemia. The average of parasitaemia was tested by Mann–Whitney U Test, which showed that the percentage of parasitaemia of those infected with *P. falciparum* is significantly higher than those with *P. vivax* (p = 0.0115). **b** miRNAs isolated from plasma-derived EVs were analysed by RT-qPCR (TaqMan™ Advanced miRNA Assays) with specific primers for hsa-miR-451a, hsa-miR-15b-5p, hsa-miR-16-5p, hsa-let-7a-5p and hsa-miR-150-5p. The plots show the average Cq values for each miRNA, and these values are compared between malaria infected patients and uninfected individuals. (Presented as standard error of mean, SEM)

57 plasma samples were included in this study divided into three groups; *P. vivax*-infected patients (n = 19), *P. falciparum*-infected patients (n = 18), and uninfected community individuals (n = 20) as per Table 2. The average age of malaria patients (Mean ± SD) was 32.68 ± 11.08 years, *P. vivax*-infected patients was 31.6 ± 9.59, and *P. falciparum*-infected patients was 33.4 ± 11.91. The average age of uninfected individuals (21.8 ± 0.89 years) was significantly lower than both *P. vivax*-infected patients or *P. falciparum*-infected patients (p = 0.018, p = 0.001, respectively). Percentage of parasitaemia (% Parasitaemia) of P. VIVAX-infected patients was 0.35 ± 0.28 while *P. falciparum*-infected patients was 1.29 ± 1.48. Overall parasitaemia was higher in *P. falciparum*-infected patients than *P. vivax*-infected patients (p = 0.0115) as shown in Fig. 1a.

**Table 2:** Sample characteristic

Characteristics	PV-infected patients	PF-infected patients	Uninfected
Total number of patients	19	18	20
Gender			
Male	16	17	11
Female	3	1	9
Age (Mean ± SD)*	31.6 ± 9.59	33.4 ± 11.91	21.8 ± 0.89
% Parasitaemia (Mean ± SD)**	0.35 ± 0.28	1.29 ± 1.48	

* Mean and SD of age *P. vivax*-infected patients was calculated from 10 patients, Mean and SD of age of PF-infected patients was calculated from 15 patients

** %parasitaemia of *P. vivax*-infected patients calculated from 16 patients. %parasitaemia of PF-infected patients calculated from 14 patients

### Relative expression of miRNAs in three biological groups

Total RNA was extracted from EVs pellets and analysed hsa-miR-451a, hsa-miR-15b-5p, hsa-miR-16-5p, hsa-let-7a-5p and hsa-miR-150-5p among *P. vivax*-infected patients, *P. falciparum*-infected patients and uninfected individuals. The average C_q_ was calculated from triplicate run (Fig. 1b). Hsa-miR-451a had the lowest C_q_ with the minimum standard deviation compared to the other miRNAs, at 26.36 ± 2.33 (Mean ± SD). A Kruskal–Wallis test was undertaken to compare the Cq values in each biological group and showed that the values were not statistically different. These results indicated that this miRNA was the most stable among three biological groups and could be used as internal control. Moreover, previous studies demonstrated that hsa-miR-451a was highly expressed in both uninfected and parasitized red blood cells [32] and its expression was independent on the intra-erythrocytic development of the malaria parasite [33].

**Fig. 2:**
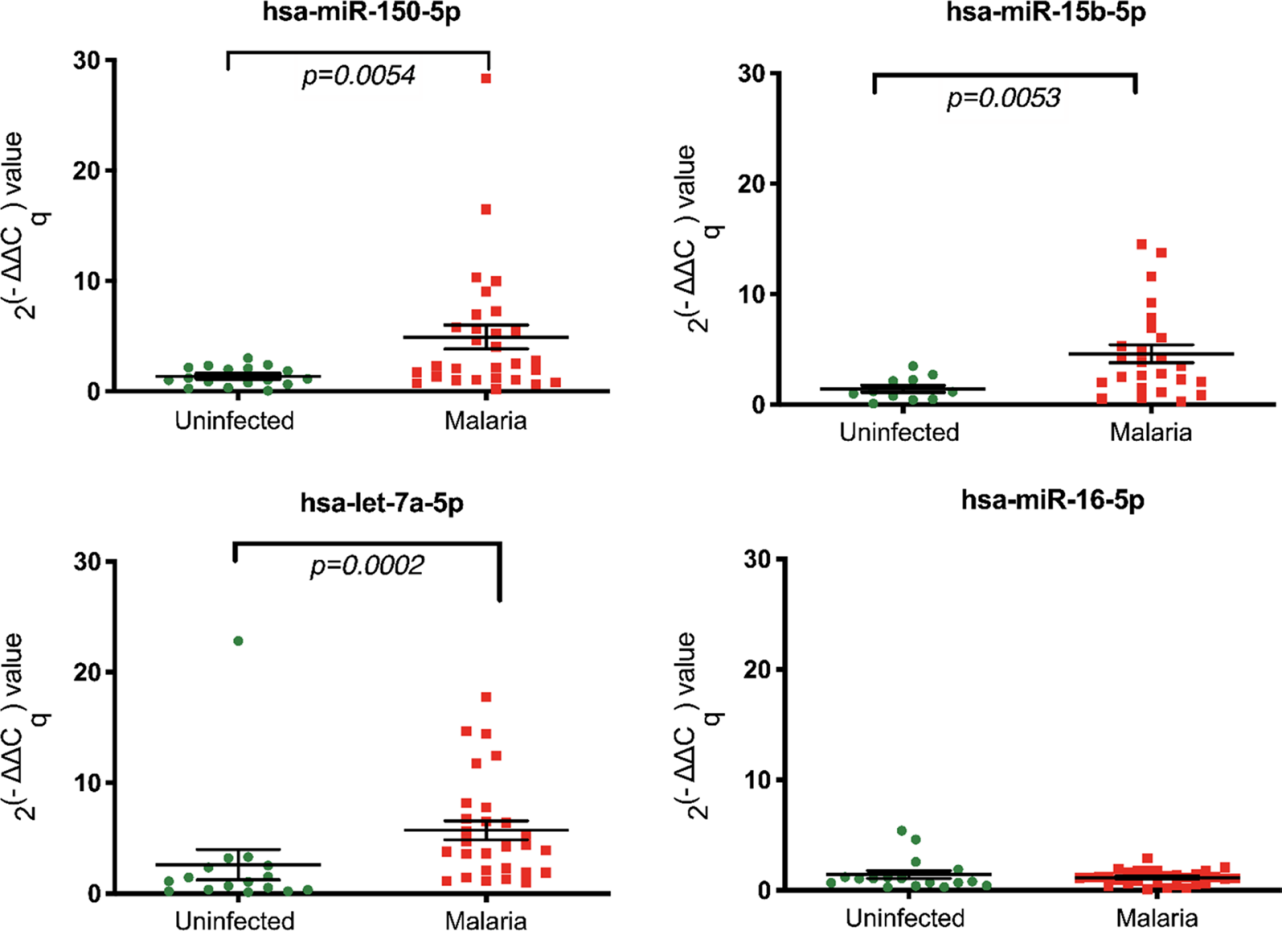
Relative expression of hsa-miR-15b-5p, hsa-miR-16-5p, hsa-let-7a-5p and hsa-miR-150-5p were calculated using 2^(−ΔΔCq)^ method. The dot plots represent 2^(−ΔΔCq)^ values of each miRNA (Mean and SEM). Mann–Whitney U Test was performed to demonstrate the different abundance of hsa-miR-150-5p, hsa-miR-15b-5p, and hsa-let-7a-5p in malaria compared to uninfected individuals (p = 0.0054, p = 0.0053, p = 0.0002, respectively). The horizontal line denotes the average value and SEM bar

In the present study, EVs were isolated from Thai patients presenting with uncomplicated malaria and demonstrated that miRNAs can be detected in these EVs. Descriptive statistics of Cq values are presented in Table 3. The relative expression of each miRNA was calculated using hsa-miR-451a as endogenous control using the 2^(−ΔΔCq)^ method [50]. The relative expressions of hsa-miR-15b-5p, hsa-miR-16-5p, hsa-let-7a-5p and hsa-let-7a-5p were compared between malaria-infected (regardless of the parasite species) and uninfected individuals (Fig. 2). Hsa-miR-150-5p (p = 0.0054), hsa-miR-15b-5p (p = 0.0053) and hsa-let-7a-5p (p = 0.0002) were significantly up-regulated in EVs from malaria-infected patients comparing to the uninfected group. However, the relative expression of hsa-miR-16-5p between infected and non-infected individuals was not different.

**Table 3:** Descriptive statistics of quantification cycle (C_q_) values

miRNAs	Min Cq	Max Cq	Mean ± SD	Coefficient of variation (%)
hsa-miR-451a	20.43	30.63	26.36 ± 2.33	8.84
hsa-miR-15b-5p	24.57	34.77	30.06 ± 2.34	7.77
hsa-miR-16-5p	22.96	33.57	29.20 ± 2.65	9.09
hsa-let-7a-5p	24.57	34.19	31.17 ± 2.15	6.91
hsa-miR-150-5p	24.07	33.54	28.78 ± 2.37	8.24

**Fig. 3:**
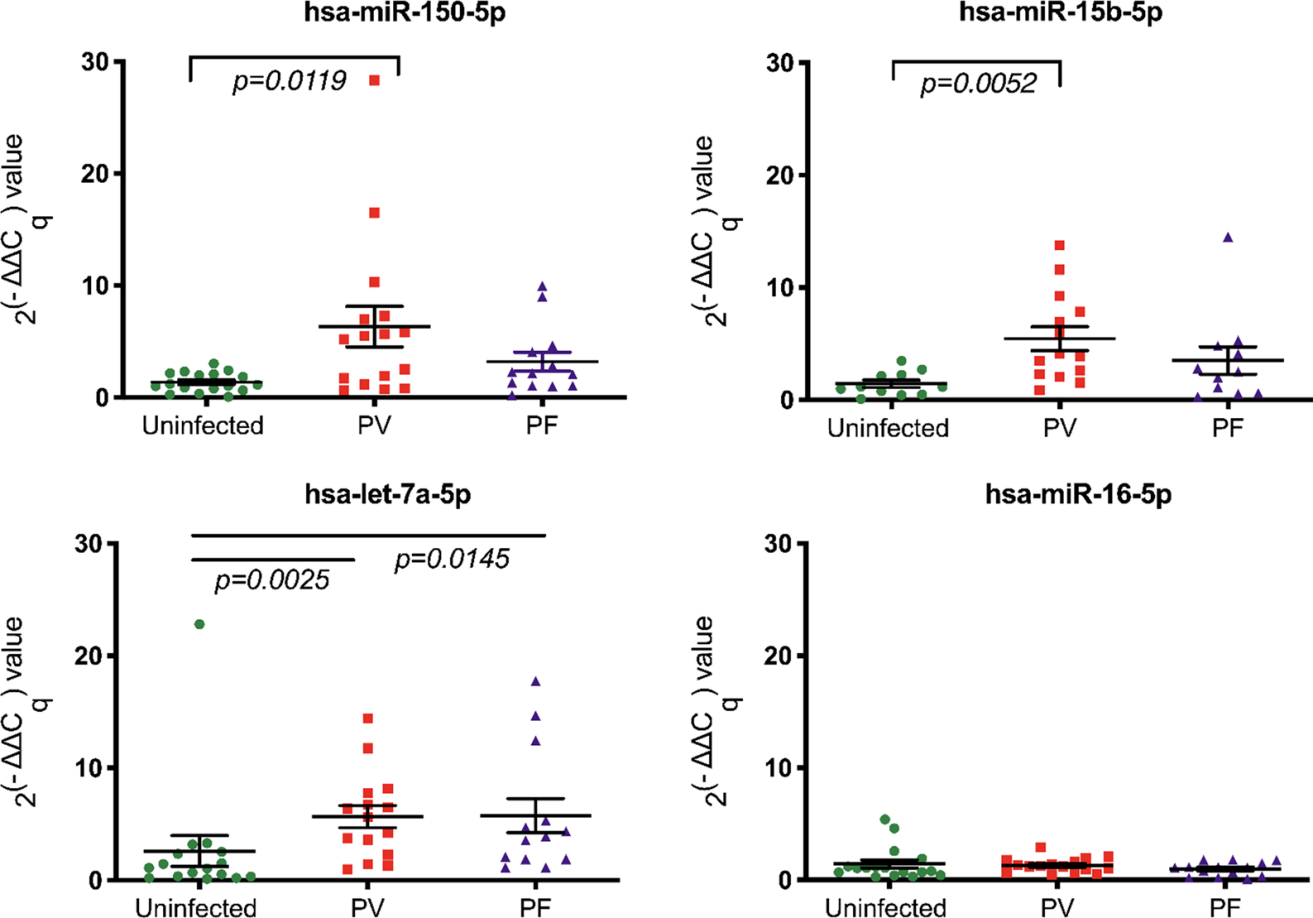
This dot plot demonstrates the miRNAs expression values in 3 biological groups (Uninfected, *P. vivax*-infected patients or *P. vivax*, and *P. falciparum*-infected patients or *P. falciparum*). The Kruskal–Wallis was tested and followed by post hoc analysis using Dunn’s test. hsa-miR-150-5p, hsa-miR-15b-5p were significantly up-regulated in *P. vivax* (p = 0.0119, p = 0.0052, respectively). Relative expression of hsa-let-7a-5p was higher in both *P. vivax* and *P. falciparum* (p = 0.0025, p = 0.0145, respectively). The horizontal line denotes the average value and SEM bar

Next, the changes in the abundance of these miRNAs were examined among the three biological groups (Fig. 3). An up-regulation of hsa-miR-150-5p (p = 0.0119) and hsa-miR-15b-5p (p = 0.0052) was observed in EVs from *P. vivax*-infected patients. Remarkably, hsa-let-7a-5p expression was higher in both *P. vivax*-infected patients and *P. falciparum*-infected patients (p = 0.0025, p = 0.0145, respectively). When those parameters were compared between *P. vivax*-infected patients and *P. falciparum*-infected patients, there was no difference. The descriptive analysis of each miRNA in each group is presented in Table 4.

**Table 4:** Relative expression analysis of miRNAs

	ΔC_q_ (Mean ± SD)	2^(− ΔΔC^_q_^)^ Mean (Mean ± SD)
hsa-let-7a-5p		
Uninfected	6.23 ± 1.91	1.28 ± 1.10
PV	4.09 ± 1.17	5.68 ± 3.85
PF	4.26 ± 1.32	5.76 ± 5.52
hsa-miR-15b-5p		
Uninfected	5.34 ± 1.49	1.43 ± 1.07
PV	3.29 ± 1.17	5.45 ± 3.96
PF	4.33 ± 1.68	3.51 ± 4.04
hsa-miR-16-5p		
Uninfected	2.79 ± 1.22	1.44 ± 1.47
PV	2.56 ± 0.72	1.31 ± 0.64
PF	3.21 ± 1.32	1.00 ± 0.60
hsa-miR-150-5p		
Uninfected	3.39 ± 1.44	1.37 ± 0.86
PV	1.54 ± 1.64	6.32 ± 7.23
PF	2.33 ± 1.49	3.20 ± 3.07

### Target prediction and KEGG Pathway analysis of hsa-miR150-5p and hsa-miR-15b-5p

In order to study the possible roles of up-regulated miRNAs in malaria patients, the prediction was made using Targetscan. These targets were later overlapped with genes involved in the biological pathways that are relevant to malaria. There were 15, 5, and 6 targets of hsa-miR-150-5p, hsa-miR-15b-5p, and hsa-let-7a-5p, respectively as shown in Additional file 1: Table S1. Some targets were regulated by more than one miRNA of interest. Hepatocyte growth factor (HGF) encoding gene was regulated by all up-regulated miRNAs. Toll-like receptor 4 (TLR4), interleukin 10, and thrombospondin-1 were found regulated by hsa-miR-150-5p and hsa-let-7a-5p.

**Fig. 4:**
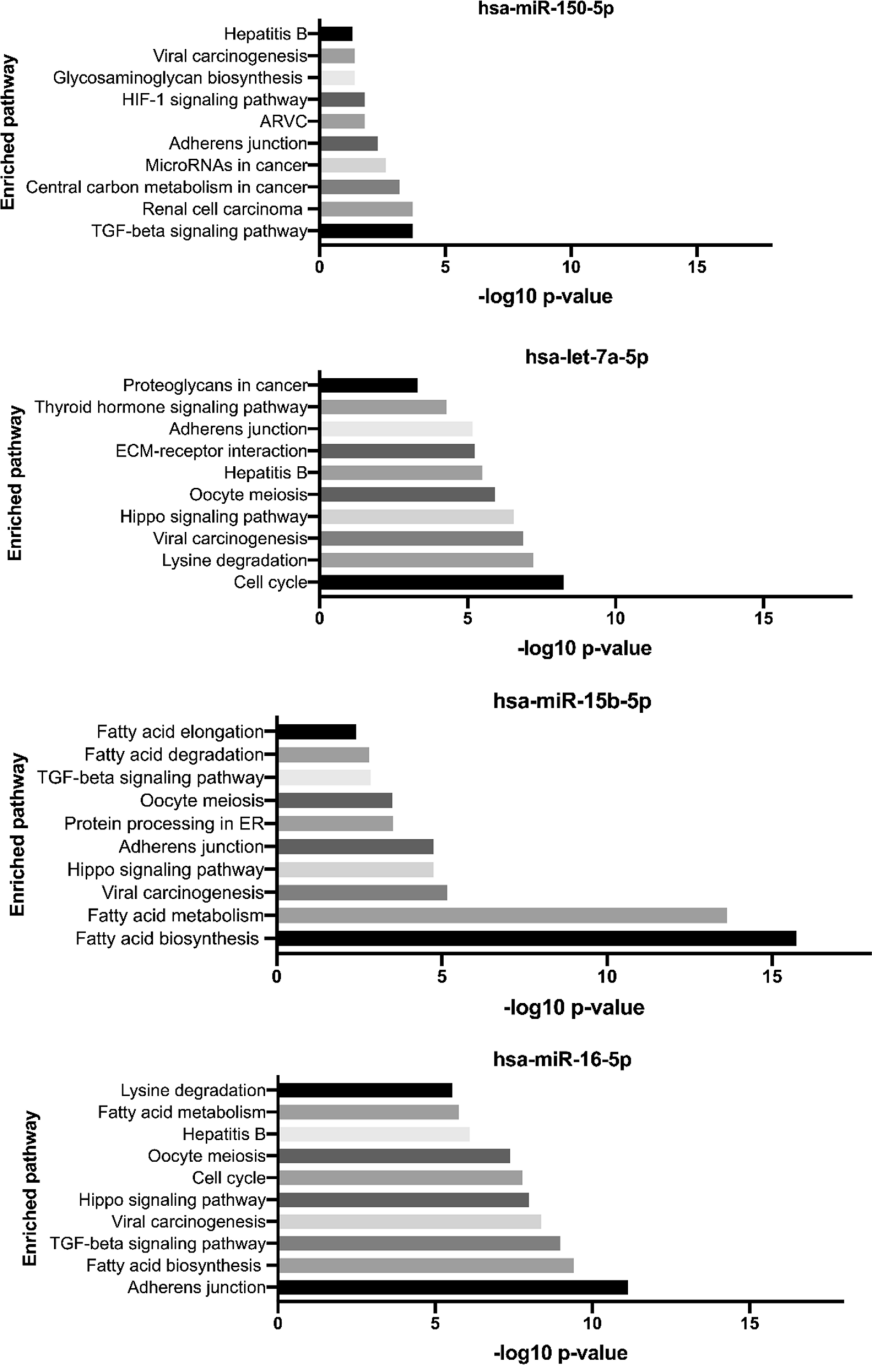
Pathway enrichment analysis of up-regulated miRNAs were carried out by DIANA-mirPath v3.0 which searching against experimentally validated miRNAs targets on Tarbase v7.0. The combinatorial effect of up-regulated miRNAs was determined. Of those enriched pathways, the malaria-relevant pathways was found including adherens junction and TGF-beta signaling pathway. The bar chart presents with top 10 enriched pathway. The horizontal lines present -log10 p-values

Next, to get insight into these up-regulated miRNAs, a KEGG pathway analysis was performed using DIANA-mirPath v3.0 that searches against experimentally validated miRNAs targets on Tarbase v7.0. The pathway analysis revealed 10, 22, and 32 pathways that were statistically overrepresented by targeted genes of hsa-miR-150-5p, hsa-miR-15b-5p, and hsa-let-7a-5p, respectively. When the combined analysis of up-regulated miRNAs was determined, there were 44 enriched pathways. Of those enrichment pathways, the overlapped pathways included adherens junction (5th in the list, p = 2.52E^−10^) as well as TGF-beta signaling pathway (11th in the list, p = 1.64E^−7^). Other important pathways that might engage in malaria were extracellular matrix (ECM)-receptor interaction (14th in the list, p = 0.0005), FoxO signaling pathway (29th in the list, p = 0.0332) and HIF-1 signaling pathway (30th in the list, p = 0.0353). In Fig. 4, the top 10 enriched pathways analysis were presented according to their ranks based on p-value.

### Evaluation of the diagnostic potential of extracellular vesicles-derived miRNAs

**Fig. 5:**
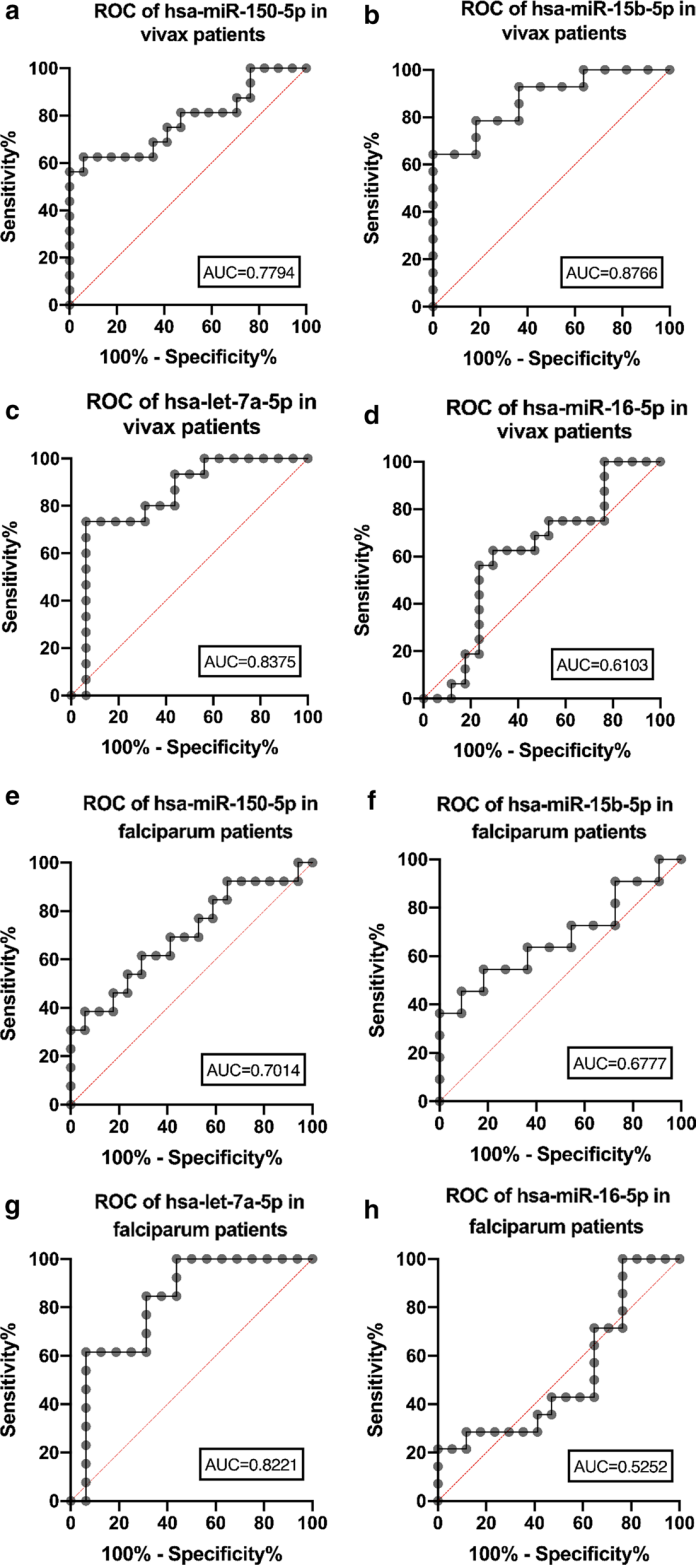
Area Under the Receiver Operating Characteristic (ROC) curve (AUC) analysis. The AUC of each tests presents with the maximum AUC. In *P. vivax*-infected patients, hsa-miR-150-5p AUC was 0.7794 (p = 0.0062), hsa-miR-15b-5p AUC was 0.8766 (p = 0.0015), and hsa-let-7a was 0.8375 (p = 0.0014). For the *P. falciparum*-infected patients, there was the hsa-let-7a-5p that was statistically significant with AUC 0.8221 (p = 0.0033)

In addition, this study aimed to investigate whether the EVs-bound miRNAs could be used as putative diagnostic markers. The ROC was used, and the AUC was calculated for each miRNA in both *P. falciparum*-infected patients and *P. vivax*-infected patients. Three miRNAs showed a statistical significance in the *P. vivax*-infected patients. hsa-miR-150-5p AUC was 0.7794 (p = 0.0062), hsa-miR-15b-5p AUC was 0.8766 (p = 0.0015) and hsa-let-7a was 0.8375 (p = 0.0014). In the *P. falciparum*-infected patients, only hsa-let-7a-5p was statistically significant with AUC 0.8221 (p = 0.0033). Area under the ROC was shown with 95% confidence interval value (Fig. 5).

## Discussion

The present study, demonstrated that miRNAs can be detected in EVs were isolated from the blood of both Thai patients presenting with uncomplicated malaria and healthy donors. The miRNAs selected included hsa-miR-451a, hsa-miR-15b-5p, hsa-miR-16-5p, hsa-let-7a-5p and hsa-miR-150-5p that were previously analysed in the context of malaria [37–39, 41–44, 48]. As mentioned earlier, hsa-miR-451a was selected as endogenous control as its Cq values were the most stable among three groups. In contrast, Chamnanchanunt et al. found that hsa-miR-451a was down-regulated in *P. vivax*-infected patient plasma [44]. Other studies demonstrated that EVs cargo hsa-miR-451a could be internalized to target the parasites and diminish the parasite burden [38, 43]. Furthermore, in an in vitro study, red blood cell derived EVs containing hsa-miR-451a and human argonaute 2 (Ago2) were shown to be internalized by endothelial cells. This miR-Ago2 could down-regulate the expression of CAV-1 and ATF2 resulting in endothelial cells alteration which is a plausible factor contributing vascular dysfunction in cerebral malaria [42]. As various studies showed that EVs that cargo hsa-miR-451a could be taken up by various cells in the context of malaria, it would, therefore, be interesting to enumerate the number of EVs and compare it with the change in abundance of hsa-miR-451a.

The hsa-miR-15b-5p and hsa-miR-150-5p were up-regulated in the plasma-derived EVs from *P. vivax*-infected patients but not in the *P. falciparum*-infected patients. An analysis of miRNAs from whole blood of adult imported falciparum malaria showed a down regulation of hsa-miR-150-5p [48]. Kaur et al. have identified a potential biomarker for differential diagnosis between uncomplicated and complicated *P. vivax* malaria infection, hsa-miR-7977. In addition, an increase in this miRNA was predicted to be involved in malaria pathogenesis through the transforming growth factor beta (TGF-β) signaling pathway [48]. Recently, a study in non-human primate (*Aotus lemurinus lemurinus*) confirmed that bone marrow is an important reservoir for gametocytogenesis and proliferation of *P. vivax* [55]. A study in bone marrow aspirate of human diagnosed with *P. vivax* showed an aberrant expression of miRNAs in CD71 positive erythroid cells during infection, hsa-miR-150 and hsa-miR-16 were down-regulated while hsa-miR-144 was increased. However, hsa-miR-451a, the most abundant erythroid miRNA, remained stable [56]. In experimental cerebral malaria (ECM), an investigation in brain tissue found a higher expression of miR-150 while microvesicles from ECM mice showed non statistically different change of miR-150 expression [39, 47]. Nonetheless, this present study only investigated the plasma from the patients before drug administration and the clinical manifestation after treatment were not followed. Taken together, these data suggest that hsa-miR-150-5p might be an essential miRNA involved in malaria infection.

The relative expression of hsa-miR-15b-5p was higher in EVs from *P. vivax*-infected patients. To date, there is no evidence about the altered expression of this miRNA which might link to *P. vivax* malaria. However, this miRNA is present in *P. falciparum* in vitro and its abundance was decreased following infection. Also, it was potentially predicted to form a chimeric fusion with ring-infected erythrocyte surface antigen (RESA) [38]. Even though the relative expression of this miRNA was not statistically significant in *P. falciparum*-infected patients, it was noticed in this study that it was slightly higher when this miRNA was compared with the uninfected group.

Next, the relative expression of hsa-let-7a, which was found in infected erythrocytes [33], was determined. The relative abundance of hsa-let-7a was significantly increased in both *P. vivax*-infected patients and *P. falciparum*-infected patients compared to uninfected controls. these results are in accordance with previous studies showing that this miRNA could be detected in EVs and might be derived from parasitized red blood cells [41, 42]. It is noteworthy that the overall expression of this miRNA is higher in the malaria patients (regardless of the parasite) than in uninfected donors. Several studies demonstrated that hsa-let-7a plays a role in host-parasite interaction [37, 41]. This miRNA in complex with Ago2 [41] could be detected in *P. falciparum* and potentially targets the *Plasmodium* gene: Rad54 [37]. In addition, hsa-let-7a expression was low in erythrocyte-derived EVs in a *P. falciparum* in vitro experiment but another member in the let7 family: hsa-let-7b was higher in the EVs fraction compared to infected or uninfected erythrocytes. Interestingly, miRNAs profiling from in vitro *P. falciparum* infected erythrocyte-derived EVs also showed that hsa-let-7a, hsa-let-7i, hsa-let-7 g and hsa-let-7f were highly expressed [42]. Some of these miRNAs might derive from parasitized red blood cells as they were found highly enriched in previous analyses [33]. In addition, a study in experimental cerebral malaria demonstrated an increased expression of let-7i in brain tissues which might link to cerebral malaria pathogenesis [39]. This implies that miRNAs within the let-7 family might play a role in the parasite biology, malaria pathogenesis and further studies should be performed to elucidate their functions.

Furthermore, the relative expression of hsa-miR-16-5p was analysed. There was no change in the abundance both in *P. vivax*-infected patients and *P. falciparum*-infected patients when compared to uninfected individuals. This is consistent with two previous studies showing that hsa-miR-16-5p is highly expressed in both *P. falciparum*-infected erythrocytes and normal erythrocytes. Thus, the relative expression of this miRNA might not be modulated during infection. However, these results differ from a study that found down-regulation of hsa-miR-16-5p in *P. vivax*-infected patients [44]. Interestingly, a previous study using an ECM model (*P. berghei* strain ANKA) found an up-regulation of miR-16 in plasma-derived microvesicles [47] suggesting that further studies are needed to elucidate the expression of this miRNA during complicated and uncomplicated malaria infection.

Several pathways might be important in the context of malaria. Importantly, the adherens junction and the transforming growth factor (TGF)-β were found enriched by the targeted genes of 3 dysregulated miRNAs. Adherens junctions are possibly regulated by hsa-miR-150-5p, hsa-miR-15b-5p and hsa-let-7a-5p. The blood–brain barrier (BBB) is a vital compartment of central nervous system as it separates the CNS from surrounding environment. Adherens junctions in endothelial cells participate to the forming and maintaining of the integrity of the BBB. A number of studies also showed that some miRNAs might regulate this type of junction. For example, the down regulation of vascular endothelium cadherin (VE-Cadherin) was affected by overexpression of miR-101 and this lead to HIV-associated neurological disorder [57]. Also, the overexpression of miR-142-3p repressed the expression of VE-Cadherin and impaired vascular integrity in zebrafish [58]. Similarly, in ECM, the authors postulated the roles of overexpressed miR-19a-3p and miR-19b-5p in this pathway as well [40]. Knowing the roles of miRNAs in the context of malaria particularly cerebral malaria pathogenesis is paramount as it might lead to the development of an adjunctive therapy. For instance, inhibition of miR-27 could prevent vascular leakage associated with ischaemia [59]. However, no study of the dysregulated miRNAs analysed in this work, which are in association with this pathway, has been performed in human malaria. More studies are therefore needed to fill this gap.

Circulating miRNAs have been studied and proposed as diagnostic biomarkers in many infectious diseases including malaria. Most studies on infectious diseases have detected human miRNAs such as those in tuberculosis [60], hepatitis B [61], schistosomiasis [62] while some studies investigated microbial miRNAs as biomarkers as well [54, 63–65]. Despite the fact that *Plasmodium spp.* lack RNA interference machinery and its own miRNAs [32, 36], human derived miRNAs were demonstrated as promising biomarkers [44, 48]. For example, in human malaria, hsa-miR-16 and hsa-miR-451a were proposed to be biomarkers for *P. vivax* infection diagnosis [44]. In the present study, the potential of miRNAs isolated from EVs in malaria patients was evaluated for the first time. The calculated p-values from AUC analysis indicated that hsa-miR-150-5p, hsa-miR-15b-5p might be used as biomarkers for *P. vivax* malaria whereas hsa-let-7a-5p might be used to test for both *P. vivax* and *P. falciparum* malaria. However, the sensitivity and specificity were not much higher. Therefore, further analysis of these miRNAs is recommended because the number of patient samples were relatively low in this study. Furthermore, as mentioned earlier, this study selected the miRNAs based on previous studies that investigated these miRNAs in the context of malaria. A more in-depth study is needed to develop new biomarkers. For instance, profiling miRNA using microarrays or next-generation sequencing will allow an evaluation of all miRNAs present in the EVs. Also, these miRNAs should be analysed in the patients after they recover from the disease. It might be useful in the context of *P. vivax* malaria as this species can cause relapse infection. In addition, these markers should be further analysed and compared in the different groups of *P. falciparum* malaria patients such as uncomplicated and severe malaria. These will be useful if patients can be early predicted the chance of developing severe malaria beforehand.

## Conclusion

This novel study explored hsa-miR-150-5p, hsa-miR-15b-5p, hsa-let-7a-5p, and hsa-miR-16-5p which were isolated from EVs from Thai malaria patient’s plasma. The relative expression of hsa-miR-150-5p and hsa-miR-15b-5p were significantly higher in *P. vivax*-infected patients where hsa-let-7a-5p was significantly up-regulated in both *P. vivax*-infected patients and *P. falciparum*-infected patients. Targets prediction and pathways enrichment analysis also provided the possible roles of these up-regulated miRNAs in the context of malaria, especially the TGF- β pathway, which need further investigation to elucidate their exact roles in the malaria biology and the disease pathogenesis.

As this current study only evaluated those miRNAs from EVs of uncomplicated malaria patients, it is therefore encouraging to analyse in the future the EVs-derived miRNAs in those patients with severe complications.

## Supplementary information

**Additional file 1: Table S1.** Individual target prediction of up-regulated miRNAs and genes involved in malaria pathway.

## Data Availability

The datasets used and/or analysed during the current study are available from the corresponding authors on reasonable request.
