# Supplementary material for: The characterization of extracellular vesicles-derived microRNAs in Thai malaria patients
Source: Malar J. 2020 Aug 10;19:285. doi: 10.1186/s12936-020-03360-z (PMC7418320; doi:10.1186/s12936-020-03360-z)
Supplement: Supplementary file 1 — Additional file 1: Table S1. Individual target prediction of up-regulated miRNAs and genes involved in malaria pathway. [file 12936_2020_3360_MOESM1_ESM.docx]

**Additional file**

**Table S1 Individual target prediction of up-regulated miRNAs and genes involved in malaria pathway**

| Up-regulated miRNAs | Predicted target genes |
| --- | --- |
| hsa-miR-150-5p | *SDC2, LRP1, HGF, GYPC, MYD88, TLR4, IL10, TGFB1*  *TNF, IL18, CSF3, KLRC4, CD36, THBS1, ITGAL* |
| hsa-miR-15b-5p | *HGF, TLR4, TGFB2, THBS2, CD40* |
| hsa-let-7a-5p | *HGF, TLR4, IL10, IL6, VCAM1, THBS1* |

**Abbreviation** *SDC2:* *Syndecan-2, LRP1:* Low density lipoprotein receptor-related protein 1, *HGF:* Hepatocyte growth factor, *GYPC:* Glycophorin C, *MYD88:* Myeloid differentiation primary response 88, *TLR4:* Toll Like Receptor 4, *IL10:* Interleukin 10,

*TGFB1:* Transforming Growth Factor Beta 1, *TNF:* Tumor necrosis factor

, *IL18:* Interleukin 18, *CSF3:* Colony Stimulating Factor 3, *KLRC4:* Killer Cell Lectin Like Receptor C4, *CD36:* cluster of differentiation 36 or platelet glycoprotein 4, *THBS1:* Thrombospondin 1, *ITGAL:* Integrin alpha L chain, *TGFB2:* Transforming growth factor-beta 2 , *THBS2:* Thrombospondin-2, *CD40:* Cluster of differentiation 40, *IL6:* Interleukin 6, *VCAM:* vascular cell adhesion molecule 1
